# Allografts and lateral extra-articular tenodesis for revision anterior cruciate ligament reconstruction: enhanced rotational stability and improved functional outcomes

**DOI:** 10.1007/s00590-023-03475-4

**Published:** 2023-01-28

**Authors:** Joan Minguell Monyart, Felipe Moreira Borim, Maria Mercedes Revertè Vinaixa, Irene Portas-Torres, Joan Pijoan Bueno, Enric Castellet Feliu, Nayana Joshi Jubert

**Affiliations:** 1 Surgery and Morphological Sciences, Universitat Autónoma de Barcelona (UAB), 08193 Bellatera, Barcelona Spain; 2 Orthopaedic Surgery Department, Knee Surgery Unit, Hospital Universitari Vall d’Hebron, Passeig Vall d’Hebron 119-129, 08035 Barcelona, Spain; 3 Reconstructive Surgery of Locomotor System Group - VHIR, Hospital Universitari Vall d’Hebron, Passeig de la Vall d’Hebron 119-129, 08035 Barcelona, Spain; 4 Bioengineering, Cell Therapy and Surgery in Congenital Malformations - VHIR, Hospital Universitari Vall d’Hebron, Passeig de la Vall d’Hebron 119-129, 08035 Barcelona, Spain

**Keywords:** Anterior cruciate ligament, Allografts, Tenodesis, Rupture, Knee

## Abstract

**Purpose:**

Multiple studies have shown higher failure rate and patient-reported outcomes to be significantly worse following revision anterior cruciate ligament reconstructive (ACLR) surgery, especially using allografts. One of the reasons being rotational instability. Because of this, augmentation with lateral extra-articular tenodesis (LET) is often considered. Good short-term results in regards to functional and perceived scores and low complication rate can be expected in revision ACLR using allografts in combination with LET.

**Methods:**

Between 2014 and 2021, 46 patients were registered for revision ACLR using allografts and extra-articular augmentation (modified Lemaire) and included in this prospective study. Patients’ demographic and clinical data were collected preoperatively, postoperatively, and during the follow-up period of 12 months.

**Results:**

Patient-reported functional outcomes were statistically significant for IKDC, Lysholm, and SF-12 physical scale (*p* < 0.05). Tegner score showed a decreased number of patients who were able to return to sport at their previous level (*p* = 0.001). Stability examination tests (Lachman and pivot-shift) showed significant improvements. Concomitant lesions were present in 76.1% of patients. Ten patients (21.7%) presented major complications, including six cases of anteroposterior instability, three cases of knee pain and one graft re-rupture.

**Conclusion:**

Revision procedures are inherently challenging with a high number of associated chondral and meniscus lesions. However, good short-term functional outcomes and enhanced rotational stability with an acceptable complication rate can be expected in most cases where revision ACLR using allografts is augmented with LET.

**Study design:**

Prospective; Case series; Level of evidence IV.

## Introduction

Reconstruction of the anterior cruciate ligament (ACLR) is one of the most common surgical procedures, with a reported failure rate of 3–14% [1]. Patient outcomes are less favorable when failures occur, and they undergo revision procedures. These procedures have higher failure rates, complications, and poor functional outcomes [2, 3]. Several factors, including recurrent instability, stiffness and pain, may lead to less-than-expected results [4].

Although the cause of rotational instability after revision ACLR is multifactorial, adding an extra-articular procedure is based on its ability to restrict rotational laxity [5]. Patient satisfaction, overall knee function, return to sports, and functional scores appear to correlate more with the restoration of rotational stability than with translational stability, making it a critical short-term to mid-term goal [6, 7]. The limited body of evidence has shown that adding soft tissue procedures may lower the risk of graft re-rupture rates and improve outcomes [8]. Additionally, graft choice for revision ACLR remains controversial. Autografts have been reported to have improved patient-reported outcomes and decreased graft re-rupture rate [3, 9]. Despite this, allografts are the selected choice in 20–51% of revision cases [3, 10], while several specific details of allografts in revision ACLR cases, such as type of tendon, sterilization method, and complications, are still missing in the literature [11].

In recent years, many studies [5, 7, 8, 12] have advocated for the critical role in rotational stability and possibly graft protection of concomitant revision ACLR and lateral extra-articular tenodesis (LET). Nevertheless, only some authors [13, 14] have prospectively evaluated and reported their results regarding objective and subjective outcomes, complications, and re-ruptures rates, none using allografts. Therefore, an analysis, specifically looking into those outcomes after combined revision ACLR using allografts and LET with a minimum follow-up of 12 months, is warranted. We hypothesized that the described combination leads to good short- to mid-term outcomes and does not have specific complications.

## Material and methods

### Patient recruitment and follow-up assessment

This study was approved by Hospital Universitari Vall d'Hebron's Ethics Committee, and patients signed informed consent before being included. All patients who underwent revision ACLR using allografts and modified Lemaire LET between November 2014 and November 2021 were screened for eligibility for this prospective study. Inclusion criteria were (1) age above 18 years and capable of giving consent for study participation; (2) patients with ACLR graft rupture diagnosed by clinical symptoms and physical exam, confirmed by magnetic resonance images (MRI). Exclusion criteria included (1) concomitant ligament injuries or coronal plane deformity; (2) incomplete follow-up and clinical data.

Patients’ demographic, clinical and radiological data were collected preoperatively, postoperatively, and during the follow-up period until 12 months postoperatively. The assessment included International Knee Documentation Committee (IKDC) score, Lysholm Knee Score, Tegner activity scale (TAS), and Short-Form Health Survey (SF-12) physical and mental. In addition, range of motion and ligament instability was assessed using the Lachman and pivot-shift test, and concomitant lesions found in radiological and arthroscopic evaluation during the primary and revision surgery were recorded.

### Surgical technique

Combined spinal anesthesia with regional nerve blockade was used. A preliminary arthroscopic inspection was performed to help diagnose and treat associated meniscal and chondral injuries. Furthermore, the size of the intercondylar fossa is evaluated, and notchplasty and osteophyte removal are done if needed to avoid impingement. Progressive drilling of the tibial and femoral tunnels with cannulated drills of different sizes until completing the debridement of the previous graft site was done. Fresh-frozen allografts were prepared; suspension systems were used for femoral fixation (TightRope® RT; Arthrex, Naples, FL); interferential screw (Biocomposite®; Arthrex, Naples, FL) and ligament staple were used for a hybrid fixation on the tibia. Lastly, a modified Lemaire LET was performed.

Patients were offered a two-stage surgery (1) if tunnel widening was so significant on both the tibia and femur that one-stage bone grafting is not feasible, usually enlarged over 14-16 mm; (2) malpositioned, which could result in tunnel overlapping; (3) arthrofibrosis; or (4) local infection. The two-stage procedure involved an initial bone grafting procedure, or in the case of infection, multiple debridements followed by bone grafting, and then an incorporation phase of 20–24 weeks, allowing the bone graft to fully heal before the subsequent second stage; CT scans at 5–6 months were performed to confirm correct incorporation.

### Rehabilitation

For the first 4–6 weeks, walking with partial weight bearing was allowed using two crutches. Patients were encouraged to perform complete knee flexion and extension. Closed kinetic chain exercises and the use of a balance board to regain proprioception were performed for the first three months, and thereafter, open kinetic chain exercises were started. Noncontact sports were permitted after 3–4 months, and a return to contact sports was allowed after 5–6 months.

### Statistical analysis

Statistical analysis was performed with statistics 26 (IBM SPSS® Statistics). Categorical variables were described with their absolute values and percentages. Quantitative variables were presented by their measures of central tendency (mean and standard deviation). Preoperative and postoperative tests were compared using paired *t* tests. Differences with *p*-values < 0.05 were considered statistically significant.

## Results

**Fig. 1 and 2:**
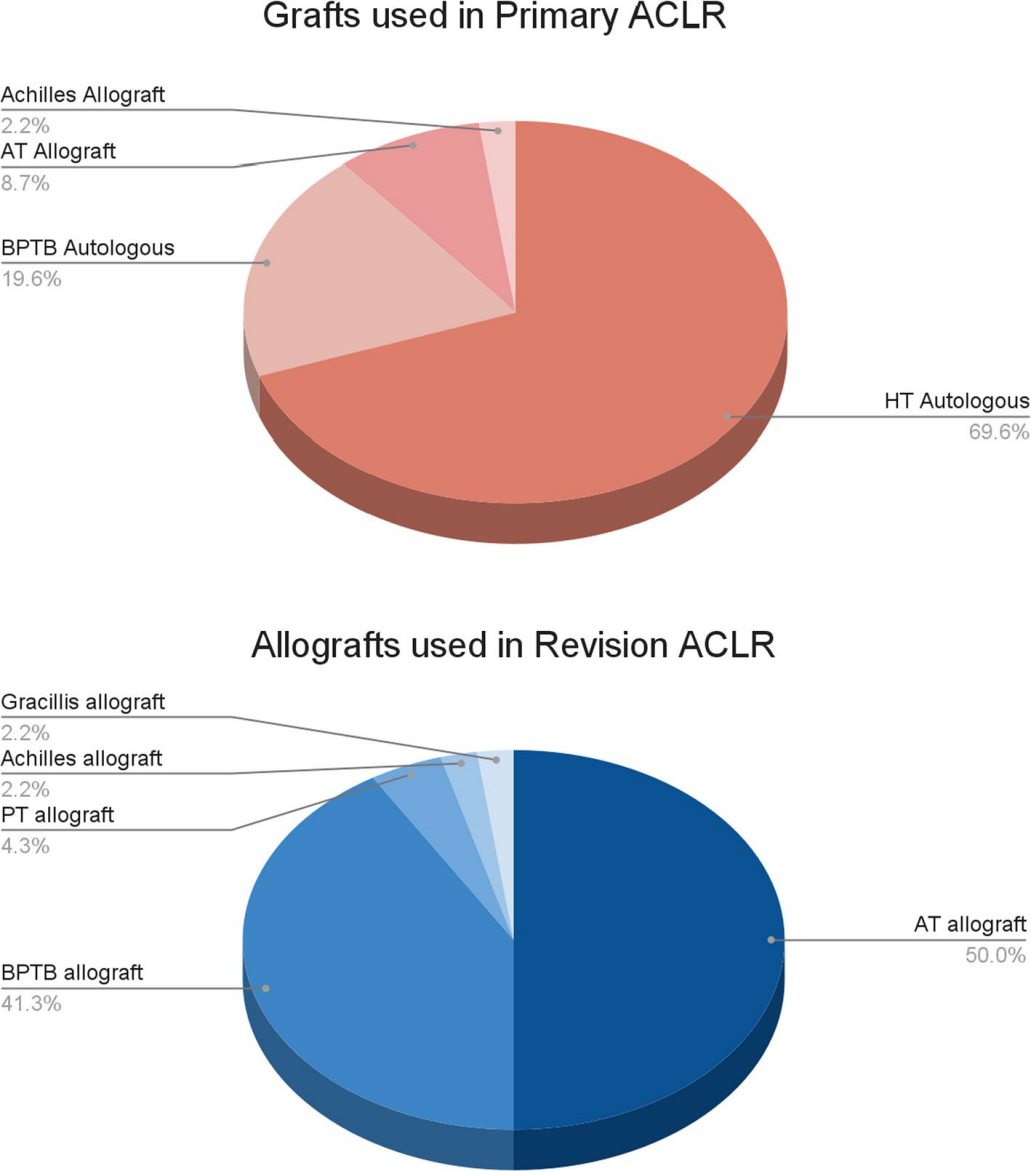
Pie charts of the grafts used in primary and revision anterior cruciate ligament reconstructions (ACLR). AT: anterior tibialis; BPTB: bone-patellar tendon bone; HT: hamstrings; PT: posterior tibialis

Forty-six patients were registered for revision ACLR using allograft and LET (modified Lemaire) and prospectively followed. Demographic and primary graft failure characteristics are summarized in Table 1. Grafts used for the primary and revision surgery are registered in Figs.  1 and 2.

**Table 1:** Demographic and primary graft failure characteristics of the included patients

*Patient data*^1^	
Sex (female/male)	15 (32.6%)/31 (67.4%)
Side (right/left)	24 (52.2%)/22 (47.8%)
Average age (SD)	36.3 (9.72)
Number of Stages (one-stage/two-stages)	34 (73.9%)/12 (26.1%)
*Primary graft failure*	
Median time (in months)^2^	58 (14.3)
One-stage	55 (12.3)
Two-stage	69 (18.1)
*Cause of primary ACLR failure*	
Traumatic event	17 (36.9%)
Technical errors	5 (10.7%)
Unknown^3^	24 (52.2%)

^1^Expressed as the number of patients and (percentage). ^2^Expressed in months and (SD). ^3^Patient did not refer to any traumatic event, nor any technical reason for failure error was detected. ACLR: anterior cruciate ligament reconstruction

Concomitant lesions were present in 76.1% of patients and are summarized in Table 2. Partial meniscectomy was performed in sixteen cases (34.8%). Notchplasty was performed in thirteen cases (28.3%) due to intercondylar notch impingement.

**Table 2:** Table with intraoperative findings of concomitant lesions

Concomitant lesions	Number	Percentage (%)
Chondral lesion (medial, lateral and femoropatellar)	22	47.8
Meniscus lesion (medial, lateral)	28	60.9
Chondral and meniscal (both)	15	32.6
Chondral or meniscal (any lesion)	35	76.1
No chondral nor meniscus lesion	11	23.9

Twelve months after the revision procedure, the functional improvement was statistically significant for Lysholm, IKDC, and SF-12 physical scales. There were no significant differences for SF-12 mental (*p* = 0.160). Tegner Activity Scale (TAS) has shown a statistically significant reduction in the activity level (*p* = 0.001). There were no professional athletes in this series, and two patients (4.3%) were associated with a player’s federation and played regularly on a weekend basis. Results are summarized in Table 3.

**Table 3:** Patient-reported outcomes summary and comparison preoperatively and after 12 months

	Preoperative	12 month follow-up	*p*
*Patient-reported*			
Lysholm^1^	27.26 (18.33)	81.57 (20.04)	0.001
TAS^2^	6.46 (1.88)	3.89 (1.73)	0.001
IKDC^3^	49.19 (13.63)	67 (18.11)	0.001
SF-12 physical^4^	43.5 (8.97)	47.81 (10.08)	0.034
SF-12 mental^4^	47.99 (11.35)	51.21 (10.31)	0.160
*Clinical evaluation*			
ROM^5^	131.56º (90º–150º)	120.54º (60º–140º)	0.001
Level of activity	Same sport, same level	Same sport, lower level	Another sport, lower level
	5 (10.87%)	25 (54.34%)	16 (34.78%)

Values for outcomes are expressed as mean and (SD). ROM is expressed as mean and (range). Level of Activity is expressed in numbers of patients and (percentages). ^1^Lysholm Knee Scoring System. ^2^Tegner Activity Scale. ^3^International Knee Documentation Committee subjective knee form. ^4^Short Form (12) Health Survey. ^5^Range of Movement

Improvement in flexion was statistically significant (*p* = 0.001), but the differences in extension were not (*p* = 0.058). See Table 3. There was a clear improvement on all stability examination tests at the one-year follow-up. During the preoperative period, thirty-four patients (73.9%) had a Lachman ≥ 2; this was the case for only six patients (13%) one year down the line (*p* = 0.024). Forty (86.9%) patients had ≥ 1 preoperative pivot-shift, being the case for only four (8.7%) after one year (*p* = 0.001). See Fig. 2 for the comparison.

Twenty cases (43.5%) presented some complications. Ten patients (21.7%) presented major complications, including anteroposterior laxity, pain, and graft failure. Minor complications accounted for twelve cases (26.1%), including acute complications such as hemarthrosis, superficial infections, and material discomfort. Anteroposterior laxity (Lachman ≥ 2) was considered a failure. Despite this, patient satisfaction and functional outcomes remained reasonable, and no savage procedure was necessary. Pain was associated with chondral and meniscus lesions in all three cases, and osteoarthritis was also seen in these patients at follow-up. One of the cases of pain due to osteoarthritis was a patient who undertook a two-stage procedure with poor results and later went on to a conversion total knee arthroplasty. The one case of detected graft failure was attributed to an initially repaired multi-ligamentous injury, requiring a second revision procedure with modest results obtained after it. See Table 4.

**Table 4:** Summarized major and minor complications

Major complications	
AP laxity (Lachman ≥ 2)	6 (13%)
Pain (Osteoarthritis)	3 (6.5%)
Graft failure and revision	1 (2.2%)
Minor complications	
Hemarthrosis	9 (19.6%)
Material discomfort	2 (4.3%)
Superficial Infection	1 (2.2%)

*AP* Anteroposterior

## Discussion

**Fig. 3:**
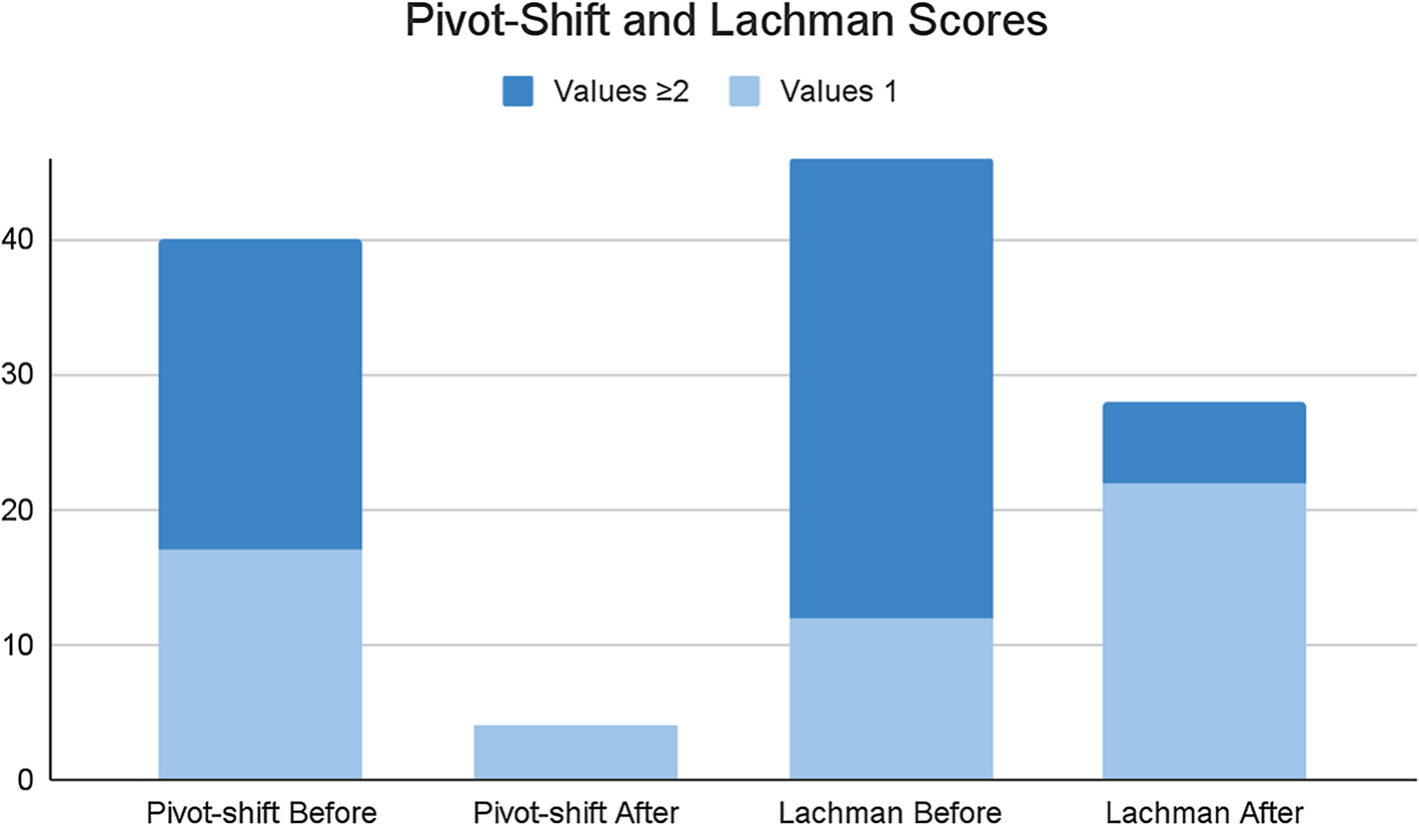
Column chart comparing pivot-shift test Lachman test scores before and after 12 months from the intervention. Values of 0 for both Lachman and pivot-shift tests were excluded from the chart for clarity

The most important findings of this study are that patients undergoing revision ACLR procedures using allografts and LET, after a one-year follow-up, had significant improvements on Lysholm, IKDC, and SF-12 physical scales. Improved residual rotatory laxity grants good short-term follow-up results with minimum re-rupture rates and acceptable rates of postoperative complications. Conversely, TAS showed a decreased level of activity. Residual anteroposterior laxity, detected by the Lachman test, appeared unrelated to poor outcomes and the need for revision (Fig. 3).

Graft choice remains controversial and thought to be implicated among the causes of revision ACLR failure [11]. Autografts have been reported to have improved sports function, patient-reported outcome measures, and decreased graft re-rupture rate at 2-year follow-up [3, 9]. Despite this, allografts are still selected in 20–51% of revision cases [3, 10], with significant improvement rates reported [11, 15, 16]. Fresh-frozen allografts offer the advantages of decreased operative times and lower morbidity, addresses the problem of limited availability of donor tissue in multiple revision cases, and in the case of bone-patellar tendon-bone (BPTB) and Achilles tendon, a bone-block can be harvested, allowing initial bony fixation and addressing the void from the index tunnel. Possible disadvantages include the risk of disease transmission, immune rejection, delay in the remodeling, and prolonged integration process [17]. Legnani et al. reported similar subjective and objective outcomes at 5.2-year follow-up when autografts and allografts were compared [15]. Grassi et al. have found autografts to have better outcomes than allografts in revision ACLR, with lower postoperative laxity and rates of complications and re-operations. However, if only non-irradiated allografts were considered, the outcomes would be similar to autografts [7]. We advocate for fresh-frozen allografts and believe that graft choice is predominantly influenced by two factors: previous graft(s) used and surgeon preference. Moreover, it is also affected by other factors, including patient preference and tunnel dilatation. The mean age of our case series was 36. 30 years, donor-site morbidity was of concern, and there was an overall low physical activity demand and sports participation in this group of patients. Graft choice in our series has gone initially from BPTB allografts with its bone stock advantage. Later, after detecting this bone stock as insufficient, we opted for tibialis anterior (TA) allografts due to their technical ease when using suspension systems with the all-inside technique.

Persistent rotatory knee laxity is increasingly recognized as a common finding after ACLR (Musahl, 2017). While the reasons behind rotator knee laxity and graft failure are multifactorial, the impact of the anterolateral knee structures appears significant. More and more studies are finding lateral augmentation to be a common indication in this scenario, with good results showing low residual rotatory laxity, low complication rate, favorable results for Lysholm, IKDC, and KOOS scores, and lowering the risk of graft re-rupture rates [7, 8, 18]. Important insights regarding extra-articular-plasty in managing failed ACLR have been provided by a few studies, such as Trojani et al., who reported a significantly higher rate of negative pivot-shift when lateral tenodesis was performed compared to isolated revision surgery [19], something similar seen in our series. Lateral augmentation procedures have shown significant heterogeneity between studies [20]. Since results across the various series published have been similar, we believe that more important than the specific lateral augmentation technique is to correctly control rotational laxity while minimizing the chances of technical errors. We have opted for a modified Lemaire LET because it is a tried and tested technique that has demonstrated to reduce anterolateral rotatory laxity and to be graft-protective [14, 21, 22].

Three studies have explicitly looked into revision ACLR and LET using modified Lemaire LET [13, 14, 23], but none used allografts. Botto et al. retrospectively reviewed eight young patients who engaged in contact sports. They stated that adding a LET helps to control rotational stability decreasing the risk of graft overstretching and re-rupture rates [23]. Lefevre et al. prospectively compared the return-to-sport rate between primary ACL reconstruction and revision surgery (fifty-five patients in this second group). They found a return-to-sport (RTS) rate of return of 87.3% for revisions. RTS at the same level showed much lower values at 12.7%. Comparatively, in our study, thirty-nine patients (84.8%) were able to RTS at a recreational level, and only five (10.9%) returned to the same sport and the same level [13]. Furthermore, Porter et al. reported that LET could neutralize persistent grade II or III rotatory laxity after isolate revision ACLR and reduce internal rotation and anterior translation using computer navigation [14]. Our results were similar; we noticed a clear improvement overall on all stability examination tests at the 12-month follow-up. During the preoperative period, forty patients had ≥ 1 preoperative pivot-shift, being the case for only four patients (8.7%) with an assessable pivot-shift of 1 after one year. Moreover, systematic reviews investigating extra-articular augmentation of ACLR have not demonstrated a reduction in rotatory laxity to be correlated with patient-reported outcome measures [21, 24]. In our case, we observed an overall improvement in stability examination tests and patient-reported scales. However, we cannot affirm it to be solely attributable to LET since correlation does not mean causation.

Since allografts have a prolonged integration process and delayed remodeling [11], LET can provide additional stability and protection against graft re-rupture during these first phases. The notion that LET may be graft-protective has previously been demonstrated by Engebretsen et al., who showed that the forces going through the ACL graft might be reduced by 43% in vitro [22]; we believe this to be crucial for the case of allograft integration in revision scenarios. Further clinical studies must be performed to understand this concept better. Only some studies have reported on allografts and LET. They advocate for the over-the-top technique, highlighting its advantage of avoiding the femoral tunnel altogether, permitting a one-stage surgery with improvements in objective and subjective scores, good RTS, and an acceptable rate of complication and failure [25, 26].

Revision ACLR procedures are known to be significantly more challenging and to present meniscal and cartilage injury in nearly 90% of patients [27]. In our case, 76.1% of all the patients had some concomitant lesions, including meniscus tears in twenty-nine patients (56.9%) and chondral lesions in twenty-four patients (47%). Surgeons must address this high prevalence of associated lesions, apprising patients of these issues before deciding on a revision procedure so that expectations can be realistic.

The complication rate in revision ACLR studies confirms the safety of combining an extra-articular procedure with intra-articular revision ACLR [7, 8]. In our series, the 21.7% complication rate was higher when compared to the 8–10% usually reported. Mainly because we considered anteroposterior laxity detected by Lachman’s test ≥ 2 (13%) to be a major complication. Despite this, patient satisfaction and functional outcomes remained favorable for these patients.

This study presents some limitations. First, it is non-comparative, with the inherent biases of this type of study. Second, our cohort is small with consistent losses, which limits its statistical power and, therefore, the generalizability of the results. Third, the outcomes are not evaluated with objective measurements, with subjective tests and scales, with 12-month follow-up. However, our study provides information on using allografts combined with LET for revision ACLR, while most studies use autografts.

## Conclusions

The use of allografts for ACL revision surgery is a safe and valid option, yielding satisfactory results regarding functional patient-reported outcomes with acceptable rates of complications. Allografts should be considered, especially in patients with low physical activity demand and when autografts cannot be safely chosen. The association of LET does not seem to increase complication rate while improving rotational stability and may be graft-protective.

## References

[1] Spindler KP, Huston LJ, Zajichek A, Reinke EK, Amendola A, Andrish JT, Brophy RH, Dunn WR, Flanigan DC, Jones MH, Kaeding CC, Marx RG, Matava MJ, McCarty EC, Parker RD, Vidal AF, Wolcott ML, Wolf BR, Wright RW, MOON Knee Group (2020). Anterior cruciate ligament reconstruction in high school and college-aged athletes: Does autograft choice influence anterior cruciate ligament revision rates?. Am J Sports Med.

[2] MARS Group (2019). Reoperation and failure rate at six years following revision ACL reconstruction: a MARS cohort study. Orthop J Sports Med.

[3] MARS Group (2014). Effect of graft choice on the outcome of revision anterior cruciate ligament reconstruction in the multicenter ACL revision study (MARS) cohort. Am J Sports Med.

[4] Ardern CL, Taylor NF, Feller JA, Webster KE (2014). Fifty-five per cent return to competitive sport following anterior cruciate ligament reconstruction surgery: an updated systematic review and meta-analysis including aspects of physical functioning and contextual factors. Br J Sports Med.

[5] Musahl V, Getgood A, Neyret P, Claes S, Burnham JM, Batailler C, Sonnery-Cottet B, Williams A, Amis A, Zaffagnini S, Karlsson J (2017). Contributions of the anterolateral complex and the anterolateral ligament to rotatory knee stability in the setting of ACL Injury: a roundtable discussion. Knee Surg Sports Traumatol Arthrosc.

[6] Ayeni OR, Chahal M, Tran MN, Sprague S (2012). Pivot shift as an outcome measure for ACL reconstruction: a systematic review. Knee Surg Sports Traumatol Arthrosc.

[7] Grassi A, Zicaro JP, Costa-Paz M, Samuelsson K, Wilson A, Zaffagnini S, Condello V, ESSKA Arthroscopy Committee (2020). Good mid-term outcomes and low rates of residual rotatory laxity, complications and failures after revision anterior cruciate ligament reconstruction (ACL) and lateral extra-articular tenodesis (LET). Knee Surg Sports Traumatol Arthrosc.

[8] Riediger MD, Stride D, Coke SE, Kurz AZ, Duong A, Ayeni OR (2019). ACL reconstruction with augmentation: a scoping review. Curr Rev Musculoskelet Med.

[9] Maletis GB, Inacio MC, Desmond JL, Funahashi TT (2013). Reconstruction of the anterior cruciate ligament: association of graft choice with increased risk of early revision. Bone Joint J.

[10] Andriolo L, Filardo G, Kon E, Ricci M, Della Villa F, Della Villa S, Zaffagnini S, Marcacci M (2015). Revision anterior cruciate ligament reconstruction: clinical outcome and evidence for return to sport. Knee Surg Sports Traumatol Arthrosc.

[11] Condello V, Zdanowicz U, Di Matteo B, Spalding T, Gelber PE, Adravanti P, Heuberer P, Dimmen S, Sonnery-Cottet B, Hulet C, Bonomo M, Kon E (2019). Allograft tendons are a safe and effective option for revision ACL reconstruction: a clinical review. Knee Surg Sports Traumatol Arthrosc.

[12] Louis ML, D’ingrado P, Ehkirch FP, Bertiaux S, Colombet P, Sonnery-Cottet B, Schlatterer B, Pailhé R, Panisset JC, Steltzlen C, Lustig S, Lutz C, Dalmay F, Imbert P, Saragaglia D (2017). Combined intra- and extra-articular grafting for revision ACL reconstruction: a multicentre study by the French Arthroscopy Society (SFA). Orthop Traumatol Surg Res.

[13] Lefevre N, Klouche S, Mirouse G, Herman S, Gerometta A, Bohu Y (2017). Return to sport after primary and revision anterior cruciate ligament reconstruction: a prospective comparative study of 552 patients from the FAST cohort. Am J Sports Med.

[14] Porter MD, Shadbolt B, Pomroy S (2018). The augmentation of revision anterior cruciate ligament reconstruction with modified iliotibial band tenodesis to correct the pivot shift: a computer navigation study. Am J Sports Med.

[15] Legnani C, Zini S, Borgo E, Ventura A (2016). Can graft choice affect return to sport following revision anterior cruciate ligament reconstruction surgery?. Arch Orthop Trauma Surg.

[16] Noyes FR, Barber-Westin SD, Roberts CS (1994). Use of allografts after failed treatment of rupture of the anterior cruciate ligament. J Bone Joint Surg Am.

[17] Cerulli G, Placella G, Sebastiani E, Tei MM, Speziali A, Manfreda F (2013). ACL reconstruction: choosing the graft. Joints.

[18] Weber AE, Zuke W, Mayer EN, Forsythe B, Getgood A, Verma NN, Bach BR, Bedi A, Cole BJ (2019). Lateral augmentation procedures in anterior cruciate ligament reconstruction: anatomic, biomechanical, imaging, and clinical evidence. Am J Sports Med.

[19] Trojani C, Sbihi A, Djian P, Potel J-F, Hulet C, Jouve F (2011). Causes for failure of ACL reconstruction and influence of meniscectomies after revision. Knee Surg Sports Traumatol Arthrosc.

[20] Nakamura N, Marx RG, Musahl V, Getgood A, Sherman SL, Verdonk P (2022). Advances in knee ligament and knee preservation surgery.

[21] Hewison CE, Tran MN, Kaniki N, Remtulla A, Bryant D, Getgood AM (2015). Lateral extra-articular tenodesis reduces rotational laxity when combined with anterior cruciate ligament reconstruction: a systematic review of the literature. Arthroscopy.

[22] Engebretsen L, Lew WD, Lewis JL, Hunter RE (1990). The effect of an iliotibial tenodesis on intraarticular graft forces and knee joint motion. Am J Sports Med.

[23] Botto G, Solessio J, Nogueira M, Alonso CL, Garate F (2016). Revisión de ligamento cruzado anterior con aumentación extraarticular de Lemaire en deportistas de contacto con alta exigencia. Reporte preliminar de casos y descripción de técnica quirúrgica Artrosc (B. Aires).

[24] Devitt BM, Bell SW, Ardern CL, Hartwig T, Porter TJ, Feller JA, Webster KE (2017). The role of lateral extra-articular tenodesis in primary anterior cruciate ligament reconstruction: a systematic review with meta-analysis and best-evidence synthesis. Orthop J Sports Med.

[25] Buda R, Ruffilli A, Di Caprio F, Ferruzzi A, Faldini C, Cavallo M, Vannini F, Giannini S (2013). Allograft salvage procedure in multiple-revision anterior cruciate ligament reconstruction. Am J Sports Med.

[26] Zanovello J, Rosso F, Bistolfi A, Rossi R, Castoldi F (2017). Combined intra- and extra-articular technique in revision anterior cruciate ligament reconstruction. Joints.

[27] Borchers JR, Kaeding CC, Pedroza AD, Huston LJ, Spindler KP, Wright RW, MOON Consortium and the MARS Group (2011). Intra-articular findings in primary and revision anterior cruciate ligament reconstruction surgery: a comparison of the MOON and MARS study groups. Am J Sports Med.

[28] Kaeding CC, Pedroza AD, Reinke EK, Huston LJ, MOON Consortium, Spindler KP (2015). Risk factors and predictors of subsequent ACL injury in either knee after ACL reconstruction: prospective analysis of 2488 primary ACL reconstructions from the MOON cohort. Am J Sports Med.

